# mHealth for Oral Care in Aging: A Narrative Review of Mobile Applications for Older Adults and Caregivers

**DOI:** 10.3390/dj14070443

**Published:** 2026-07-15

**Authors:** Mireya Martínez-García, Guadalupe Gutiérrez-Esparza, Socorro Aída Borges-Yañez, Enrique Hernández-Lemus

**Affiliations:** 1 Dental Public Health Department, Division of Graduate Studies and Research, School of Dentistry, Universidad Nacional Autónoma de México, Mexico City 04510, Mexico; aborges@unam.mx; 2 “Researcher for Mexico” Program, Secretaría de Ciencia, Humanidades, Tecnología e Innovación (SECIHTI), Mexico City 03940, Mexico; ggutierreze@conacyt.mx; 3 Diagnostic and Treatment Services, Instituto Nacional de Cardiología Ignacio Chávez, Mexico City 14080, Mexico; 4 Computational Genomics Division, Instituto Nacional de Medicina Genómica, Mexico City 14610, Mexico

**Keywords:** mHealth, oral health, older adults, caregivers, teledentistry, geriatric dentistry

## Abstract

Population aging, persistent oral health inequalities, and growing reliance on informal caregiving are creating new challenges for oral healthcare systems worldwide. Mobile health technologies (mHealth) are increasingly being used to support oral health promotion, self-care, monitoring, caregiver assistance, and workforce training. However, it remains unclear whether existing applications adequately address the needs of older adults and their caregivers. This narrative review examined the current landscape of mHealth applications for oral health in aging populations, with particular attention to tools supporting self-care, clinical monitoring, caregiver assistance, and primary healthcare training. Following the SANRA framework, we reviewed studies published between January 2000 and May 2026 and analyzed their functionalities, target users, usability, and the evidence supporting their development and implementation. Applications for oral health education and literacy were the most frequently reported. In contrast, relatively few tools were developed specifically for caregivers, dependent older adults, or individuals with cognitive impairment. Cultural and linguistic adaptation was also uncommon. Across categories, most applications had been evaluated primarily through usability or feasibility studies, with limited evidence regarding clinical effectiveness, long-term outcomes, or integration into routine healthcare services. Viewed in the context of the WHO Decade of Healthy Ageing 2021–2030 and the Global Strategy and Action Plan on Oral Health 2023–2030, these findings suggest that while mHealth may contribute to improving oral healthcare for older adults, important gaps remain in clinical validation, accessibility, and adaptation to diverse populations and care settings.

## 1. Introduction

Population aging has become one of the most significant demographic transformations of the 21st century, with profound implications for health systems worldwide [1,2,3]. By 2050, the global population aged 60 years and older is expected to surpass two billion, with the most rapid growth occurring in low- and middle-income countries. As longevity increases, so does the burden of chronic conditions, including oral diseases, such as dental caries, periodontitis, tooth loss, xerostomia, and oral mucosal lesions [4,5,6]. These conditions will affect oral functions such as chewing, swallowing, nutrition, and even, in extreme cases, speech. They also have a negative impact on quality of life, may contribute to frailty, and can adversely affect overall health [7,8,9,10]. Although oral diseases are largely preventable, they remain highly prevalent and frequently untreated in older adults. This is in particular the case of individuals with functional dependence, such as those with cognitive decline, or limited access to dental care [11,12,13,14,15].

As their functional limitations grow with age, a large proportion of older adults need to rely on both formal and informal caregivers to help them with their daily activities, including oral hygiene [16]. Family members, friends, or community members (collectively known as informal caregivers) often provide the largest share of long-term care; however, informal caregivers frequently lack training in oral health assessment and hygiene practices [17,18]. Similarly, trained professionals like formal caregivers and primary healthcare providers often show insufficient knowledge or skills to detect early signs of oral disease or to support consistent oral care [19,20,21,22,23]. These gaps often result in poor oral hygiene practices, delayed disease detection, and poorer oral health outcomes among older adults [24]. Furthermore, in scenarios with limited education, deficient access to instructional resources and structural barriers partially precluding the availability of dental care, existing inequities in oral health, in particular in resource-limited contexts may be further exacerbated. In recent years, tools such as mobile health technologies (mHealth) have been developed to help address some of these challenges [25,26,27,28,29,30,31].

**Figure 1:**
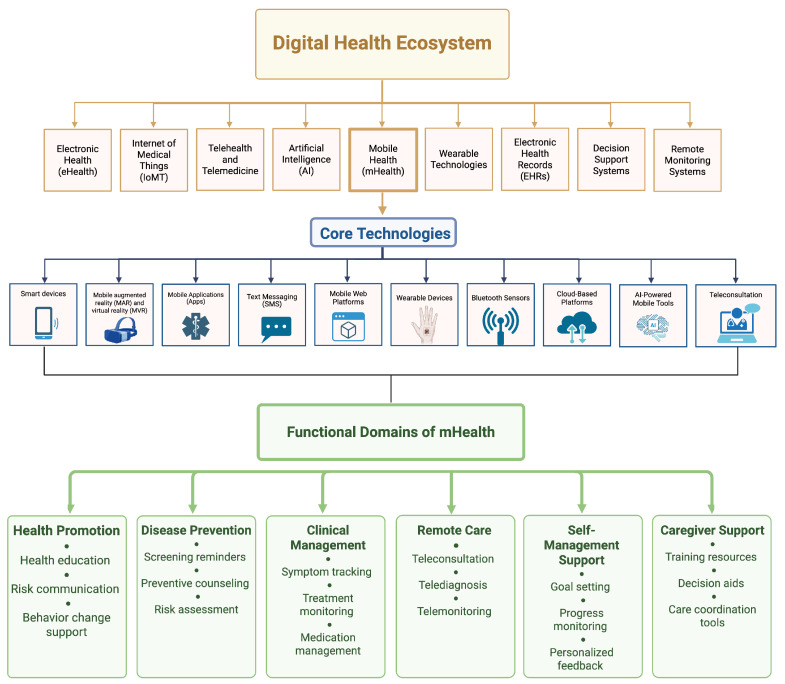
Hierarchical framework of digital health technologies. Created in https://BioRender.com.

Within the evolving digital health ecosystem, mHealth integrates mobile applications, messaging systems, wearable devices, teleconsultation platforms, cloud-based services, and other connected tools designed to extend healthcare beyond traditional clinical environments. As illustrated in Figure 1, mHealth occupies an important position within the broader digital health landscape and encompasses multiple core technologies that frequently operate in combination rather than as isolated components [32].

It is estimated that about 80% of the world’s population owns a mobile phone, although rates vary by country and region [33]. Smartphones are becoming pervasive in our societies; digital literacy and routine use are becoming increasingly common, even among older adults and caregivers, creating opportunities to support oral healthcare through mobile applications [34,35,36]. In the context of dentistry, in particular, mHealth has been used for various purposes including oral hygiene guidance, caries risk assessment, periodontal monitoring, teledentistry, and strengthening patient engagement [37,38,39,40,41].

Despite this growing interest, mobile applications specifically designed to meet the needs of older adults or their caregivers remain relatively uncommon, leaving many of the functional, cognitive, and sensory challenges associated with aging insufficiently addressed [42]. Furthermore, the quality, usability, cultural relevance, and validation of available apps are extremely heterogeneous, with a small number of well-developed tools coexisting alongside many applications with limited scope and supporting evidence. This variability makes it difficult for clinicians, caregivers, and policymakers to identify apps that are both reliable and evidence-based [33,43].

Given these limitations, a comprehensive and up-to-date synthesis of mOralHealth applications for older adults and their caregivers is warranted. The present review was undertaken to identify, describe, and critically examine existing mOralHealth technologies, together with the evidence available regarding their development, implementation, usability, effectiveness, and integration into oral healthcare. This review was conducted within the context of broader global health policy initiatives, including the World Health Organization’s (WHO) Decade of Healthy Ageing (2021–2030) [44,45,46,47], the Global Strategy and Action Plan on Oral Health 2023–2030 [48], and the mOralHealth programme developed through the International Telecommunication Union (ITU) Be He@lthy Be Mobile (BHBM) initiative [33]. These frameworks informed the interpretation of findings and the identification of current gaps and future priorities, particularly regarding equitable access to care, support for caregivers, integration of oral health into primary care, healthy ageing, and the use of digital technologies to strengthen oral health services. Regional initiatives promoted by the Pan American Health Organization (PAHO) were also considered when discussing challenges and opportunities relevant to Latin America [49,50].

These guidelines and initiatives share the following common goals to improve the oral health outcomes for aging populations:

1. Promoting equitable access to care.
2. Reaching populations in remote or resource-limited settings.
3. Supporting caregivers in developing knowledge and skills.
4. Empowering individuals to make informed health decisions.
5. Strengthening primary healthcare models.
6. Ensuring person-centered care.
7. Expanding the use of digital health technologies.

Furthermore, this review examines the current mHealth landscape aiming to promote the development of tailored interventions (i.e., ones taking regional cultural and contextual factors into account) for Latin America, since persistent structural barriers to dental services as well as a strong reliance on informal caregiving commonly create complex challenges.

**Figure 2:**
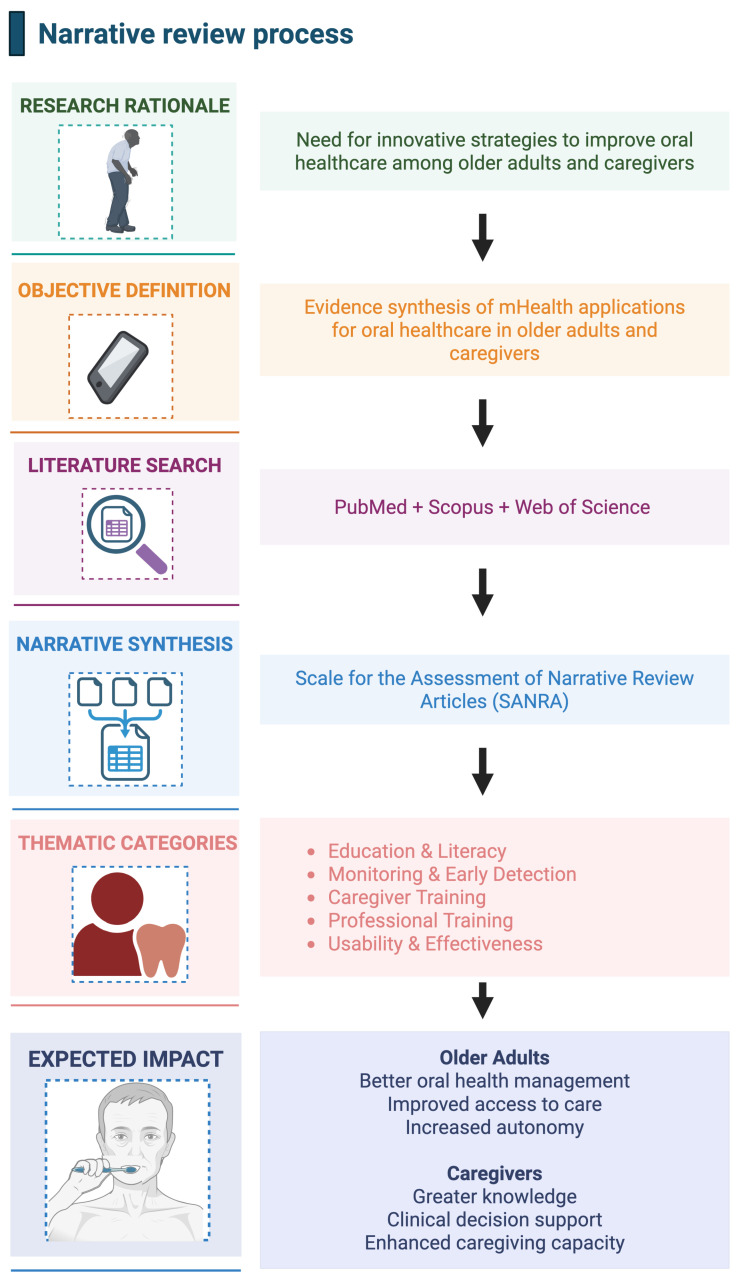
A schematic workflow for this study. Created in https://BioRender.com.

Accordingly, this narrative review seeks to identify, describe, and critically examine mOralHealth applications developed for oral health promotion education, monitoring, and caregiver training in older adults. The analysis focuses on the range of applications currently available, their intended users and core functionalities, the evidence underpinning their effectiveness, their usability and accessibility for aging populations, and the degree to which they respond to the educational needs of both formal and informal caregivers. This review also describes current opportunities and limitations, and outlines future directions for mHealth solutions in geriatric oral care (for a simplified workflow of this work, please refer to Figure 2).

## 2. Methods

### 2.1. Type of Review

This work was conducted as a narrative review. We followed the methodological principles outlined in the Scale for the Assessment of Narrative Review Articles (SANRA) guidelines [51]. The narrative approach adopted here allows a broader and more flexible engagement with the literature and is particularly well suited to integrating diverse forms of evidence, including conceptual advances, technological developments, and emerging trends. These characteristics are especially relevant for complex and rapidly evolving topics such as mHealth applications for oral care in aging populations.

### 2.2. Search Strategy

A systematic and comprehensive literature search was conducted to identify published studies on mobile health applications designed for oral health promotion, assessment, self-care, caregiver support, or training of healthcare professionals involved in the care of older adults. The search was conducted using four major scientific databases: PubMed, Scopus, Web of Science and Google Scholar. The search strategy combined controlled vocabulary and free-text keywords related to oral health, dentistry, geriatric dentistry, mHealth, mobile applications, smartphone apps, digital health, caregivers, caregiver training, informal care, long-term care, older adults, aging, and gerontology.

Eligible articles described the development, validation, usability testing, feasibility assessment, or implementation of mOralHealth applications. The review distinguished between the characteristics of the applications themselves, such as target users, functionalities, and intended purpose, and the evidence available to support their development, implementation, or evaluation. Studies were required to address at least one aspect of oral health practice or promotion, such as self-care, clinical evaluation, health education, disease prevention, teleconsultation, or monitoring. The populations of interest were older adults aged 60 years or older, caregivers of older adults (formal or informal), and primary healthcare providers involved in geriatric oral care. A broad range of study designs was considered, including development studies, pilot trials, observational research, qualitative and mixed-methods studies, and usability or feasibility assessments. Publications were limited to studies published between January 2000 and May 2026, available in full text in English or Spanish.

#### Data Synthesis

A thematic synthesis approach was used [52]. The included literature was categorized into five thematic areas: (1) mobile applications for oral health education and literacy in older adults, (2) mobile applications for surveillance, assessment, and early detection of oral health, (3) mobile applications for training formal and informal caregivers, (4) mobile applications for training primary healthcare personnel and dental providers, and (5) evaluation of effectiveness, acceptability, and usability. The findings were interpreted in the context of relevant international policy frameworks [33,44,45,46,47,48,49,50].

## 3. Findings

**Figure 3:**
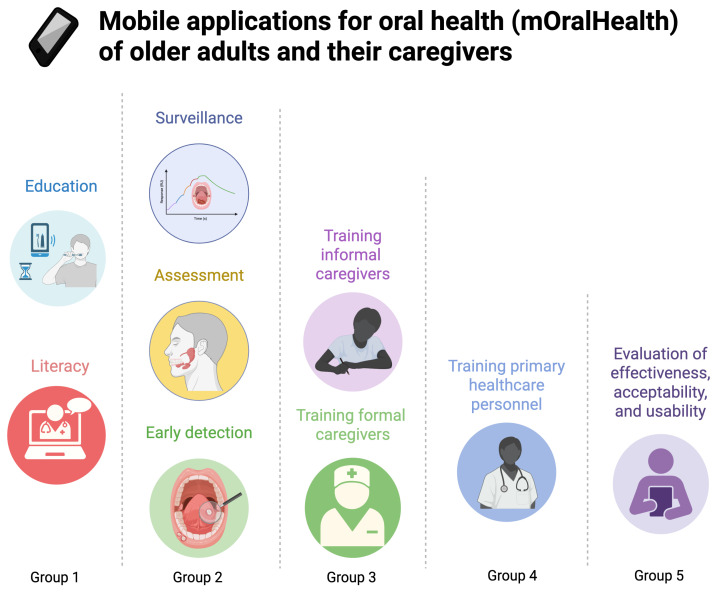
Thematic groups identified in the narrative review of mHealth applications for oral health in aging populations. Created in https://BioRender.com.

The search identified a heterogeneous body of literature addressing the development, evaluation, and implementation of mHealth applications for oral health promotion, monitoring, caregiver support, and geriatric oral care. The included studies varied considerably in their methodologies, target populations, and technological characteristics. Despite this diversity, the evidence could be organized into five thematic categories that reflect the principal areas of activity within the current mOralHealth landscape for older adults and caregivers (Figure 3). Representative applications, together with their target users, key functionalities, study designs, and evidence base, are summarized in Table 1.

### 3.1. Mobile Applications for Oral Health Education and Literacy in Older Adults

Many applications identified in the review were specifically designed to promote oral health education and literacy among older adults, with particular emphasis on disease prevention, swallowing-related quality of life, and home-based self-care strategies [53,54,55]. These tools often offer detailed instructions for hygiene practices such as brushing and interdental cleaning illustrated by animated demonstrations or short instructional videos [56,57].

These applications provided information on risk factors for dental caries and periodontitis, dietary recommendations regarding cariogenic food exposure, as well as management strategies for chewing efficiency and oral function [58]. Only a few applications offered personalized recommendations or data-driven advice tailored to the individual clinical state of the users [59].

Some of these applications were designed to improve oral health literacy by defining key concepts with common language, providing guidance for recognizing and understanding common symptoms [38]. Some interventions further integrated mobile virtual reality (MVR) and augmented reality (MAR) technologies to enhance oral health education and literacy through interactive and visually guided learning experiences tailored to older adults [32,60,61]. Most applications have simplified interfaces with large icons, minimal text, and are easy to navigate [40].

A widely used example of this approach is Brush DJ, a free mobile application developed in the United Kingdom to support oral hygiene behaviors through an intuitive interface, music-assisted toothbrushing, visual guidance, and personalized reminders for preventive oral care activities [62]. The app incorporates evidence-based content drawn from the Public Health England document Delivering Better Oral Health, as well as links to animated instructional videos and NHS health promotion resources. In a cross-sectional user perception study with 189 respondents across 182 countries, 88% reported brushing for longer, 70% perceived cleaner teeth, and 92.3% would recommend the app, suggesting high acceptability and potential benefits for motivation, education, and oral hygiene adherence [63]. The authors of DJ App acknowledge the preliminary nature of the study and the absence of clinical outcome data (see Table 1).

Another notable example is TEGO (Geriatric Dental Specialties Tele-platform) application, a Chilean educational platform designed to promote oral health and self-care among adults aged 60 years and older. It represents one of the first mOralHealth initiatives developed specifically for older adults in Chile and Latin America (Table 1). The free app offers interactive content to communicate various aspects of oral cancer prevention, dental and denture hygiene, nutrition, swallowing, and artificial saliva preparation [64].

Beyond education, TEGO integrates a teleconsultation system supported by a mobile dental unit equipped with digital technologies, including intraoral scanning and clinical imaging, allowing patient information to be uploaded to a virtual 3D oral model and reviewed remotely by multidisciplinary specialists. In a pilot implementation involving 135 Chilean older adults (mean age 72 years), user satisfaction was assessed among 68 participants. More than 75% of respondents reported positive perceptions of dental care, quality of treatment, platform usability, and willingness to recommend the service. Although clinical outcomes were not assessed, the findings suggest that TEGO is a feasible and highly accepted model for combining oral health education, remote specialist support, and mobile dental care for older adults, particularly in underserved communities [64].

In contrast, the Oral Self-Care App (OSCA) is one of the few apps with documented clinical outcome data in a predominantly middle-to-older adult population. OSCA was developed at Chang Gung Memorial Hospital in Taiwan, specifically to improve oral hygiene of patients with periodontal disease (Table 1). Clinical outcomes were assessed using the O’Leary Plaque Control Record (PCR) at baseline and re-evaluation after 4–8 weeks of periodontal initial therapy. Significant improvements in oral hygiene were observed in the intervention group compared with controls, oral hygiene differed significantly across low-, moderate-, and high-frequency users (*p* < 0.05 and *p* < 0.01 respectively), with high-frequency users showing substantially greater improvement. Overall likeability of the OSCA was high (mean 5.85/7) and strongly correlated with frequency of use (r = 0.745; p<0.001) [65].

Similar educational approaches were reported by Ki et al. (2021) and Ng et al. (2021) [55,56]. Ki and collaborators developed a mobile application OHEMA (Oral Health Education using a Mobile App). OHEMA is a mobile app-based educational intervention targeting older adults (Table 1). In a randomized controlled trial, the authors evaluated the app’s performance assessing oral function and quality-of-life outcomes. Results demonstrated statistically significant improvements in the intervention group for tongue pressure (17.75 to 27.24 kPa; p<0.001), unstimulated salivary flow rate (4.02 to 7.19 mm; p<0.001), subjective oral dryness (30.75 to 18.50; p<0.001), and swallowing-related quality of life as measured by the SWAL-QoL scale (152.10 to 171.50; p<0.001), with no significant changes observed in the control group [55].

Ming Ng and coworkers developed the Oral and Denture Guide, a bilingual progressive web application designed to provide oral hygiene instructions and illustrative demonstrations for a number of hygienic procedures to older adults. In RCT involving 52 participants, both the web-based and conventional education groups showed significant improvements in oral health knowledge and reductions in plaque-related outcomes (p<0.001). Participants using the web application achieved significantly lower gingival index scores after three months (p=0.008). User acceptability was high, with 92% of participants accessing the platform and 75% recommending it to others [56].

Along the same lines, the MAR-integrated Oral Health Education App was developed in Taiwan as an interactive educational tool for community-dwelling older adults (Table 1). Delivered through the MAKAR platform, it combines augmented reality and 3D animated content to promote oral hygiene practices and oral health literacy [60]. In a three-arm RCT involving 61 older adults (MAR: *n* = 20, lecture-based education: *n* = 22, control: *n* = 19), both intervention groups showed improvements in oral health status indicators, whereas significant gains in oral health knowledge (p=0.002) and self-efficacy (p=0.001) were observed only in the MAR group. Despite these promising outcomes, usability scores remained below the recommended threshold, highlighting persistent challenges related to technology adoption among older users [61].

Recent evidence from South Korea further supports the educational and preventive potential of smartphone-based interventions for older adults. Lee et al. (2023) [57] reported significant improvements in oral health knowledge, oral health perception, plaque control, and tongue coating following a five-week app-based education programme, while Jung et al. (2024) [66] found significant gains in anterior tongue strength and salivary flow using a smartphone-delivered oral and whole-body exercise programme. Together, these randomized trials suggest that mobile applications can support both oral health literacy and oral functional health in community-dwelling older adults, although longer follow-up periods are needed to determine the durability of these benefits [57,66].

Overall, the evidence suggests that mOralHealth applications can complement traditional patient education by improving access to oral health information and enhancing users’ understanding of dental care [67]. However, further research is needed to determine their long-term effectiveness and their impact on oral health literacy, behavioral outcomes, and clinical indicators in older populations.

### 3.2. Mobile Applications for Surveillance, Assessment and Early Detection of Oral Health

Another important group of mOralHealth applications focuses on remote surveillance, self-assessment, early detection, and digital support for oral health evaluation [40,68]. Several studies described tools that allow its users to take intra-oral pictures using a smartphone camera [69,70,71]. These images may be evaluated by dentists for remote consultation [72,73]. These images can also be used to monitor changes over time, or analyzed using image-recognition algorithms to provide preliminary evaluations [37]. However, the accuracy and usefulness of these features were often constrained by the quality of the photographs taken by older adults, as well as by inconsistencies in lighting, angle, and positioning during image capture.

Several of these applications provide guidance on selecting appropriate oral care products; others also provide a structured guide for performing oral self-examination [60]. These tools instruct users on how to identify persistent ulcers, erythematous or leukoplakic lesions, as well as signs of gingival bleeding and chewing discomfort [74]. However, although these self-examination modules were generally well received and accepted by the users, they have rarely been validated against clinical reference standards. This limits their reliability for clinical decision making or early disease detection [75].

A smaller group of applications focuses on oral hygiene assessment, early detection, and functional aspects of oral health, including perceived salivary flow, loss of chewing ability or chewing discomfort, swallowing difficulty, and dryness of the oral mucosa [66,76]. These features may be useful within community-based screening campaigns or as auxiliary tools for home-based surveillance among frail or dependent older adults [77]. These tools may complement to traditional clinical evaluations with accessible, user-friendly functional evaluations [78,79].

This approach is illustrated by HAHA2022, a mobile application developed as a digital health coach to accompany older adults with chronic diseases in their oral health day to day management [80]. The application was developed in South Korea by Park and colleagues. Usability was measured using the Korean version of the Mobile Application Rating Scale and was determined to be highly acceptable and feasible for use by this population in a mixed evaluation strategy involving both general population adult subjects and expert panel members. The authors suggest that the age-friendly features and digital health coaching capabilities will improve its adoption and ongoing use and in this way potentially helping to reduce digital and oral health disparities.

A related application is iGAM, developed by researchers at the Faculty of Dental Medicine of the Hebrew University-Hadassah in Jerusalem, Israel, and designed to detect the exact stage of gingivitis and monitor it using dental photographs taken by the patient (Table 1). Users receive a mouth-opening device and follow standardized instructions after downloading the app. They then follow the instructions to take a photograph of their gums, which are then remotely evaluated by a dentist using the Modified Gingival Index (MGI) [81]. The authors acknowledge that the app may not be directly applicable to older adults or individuals with low digital literacy, and call for future randomized trials in broader populations [82]. iGAM app is available for both Android and iOS devices. The application may also be used with caregiver assistance when needed.

Another example is MeMoSA (Mobile Mouth Screening Anywhere). This application was developed as a smartphone-based platform for standardized oral screening, photographic documentation, and remote specialist consultation. In a community screening programme conducted in rural Malaysia, all 288 images captured were considered diagnostically interpretable, participant acceptance reached 100%, and 81.3% of clinicians indicated they would recommend the platform. Despite challenges related to examination time, referral follow-up, and integration into existing health systems, MeMoSA demonstrated the feasibility and acceptability of smartphone-based oral screening in underserved settings, highlighting its potential value for older adults with limited access to specialist care [83].

In a comparable approach, the Tel-e-dent platform enabled remote oral health assessment in nursing home residents through intraoral video recordings reviewed by dental specialists (Table 1). In a multicenter study involving 235 institutionalized older adults (mean age 84.4 years), the system demonstrated excellent diagnostic accuracy for dental pathology (sensitivity 93.8%, specificity 94.2%, AUC = 0.95), while reducing examination time compared with conventional assessments (12 vs. 20 min). High acceptability (95.3%) and strong performance for evaluating prosthetic status and chewing ability support its use as an effective teledentistry tool for oral health surveillance in frail older adults with limited mobility [84].

Similar results were reported by Pandey et al. (2023) [85], who evaluated smartphone-based teledentistry for caries detection in older adults in India. Remote assessment of intraoral photographs achieved excellent diagnostic agreement (mean Cohen’s kappa = 0.909), sensitivity above 91%, and specificity above 98%, reinforcing the growing evidence that teledentistry can provide reliable and accessible oral health screening for older populations with restricted access to conventional dental care [85].

Beyond telediagnosis, mobile technologies have also been applied to oral health surveillance. Tooth Memo (formerly Oral Health Survey Mobile Application, OHSMA), developed at Chulalongkorn University in Thailand, is a mobile application designed to digitize oral health surveys and dental records using WHO-standardized examination protocols (Table 1). In a validation study involving 103 patient records, the app eliminated missing data, reduced processing time by more than three hours compared with paper-based methods, and demonstrated excellent reliability (Fleiss’ kappa = 0.93; Cohen’s kappa = 0.98). These findings highlight its potential to support large-scale oral health monitoring and epidemiological surveillance, including community-based programmes targeting older adults [86,87].

Similarly, the Saudi Electronic Caries Assessment Tool (SECAT) was developed to support standardized, WHO-based caries surveillance in remote and resource-limited settings (Table 1). The application operates offline and demonstrated high usability among clinicians, with an overall satisfaction rate of 82% and 78% reporting ease of use. These features make SECAT a useful tool for community-based oral health surveillance, including programs targeting underserved older populations [88].

Taken together, the mOralHealth technologies reviewed in this category reflect a shift from information delivery toward the collection and management of clinically relevant oral health data. Through approaches ranging from remote image capture to digital surveillance systems and specialist consultation platforms, these technologies broaden the possibilities for monitoring oral health across diverse care settings and aging populations.

### 3.3. Mobile Applications for Training Formal and Informal Caregivers

An additional group of apps was designed with caregivers in mind as they play a central role in supporting oral hygiene and the management of oral emergencies or traumatic dental injuries in dependent older adults [89,90]. Some of these tools were particularly focused on dementia care and offered simplified routines adapted to cognitive impairment. They also incorporated behavioral strategies to manage resistance during oral hygiene activities. Others include short instructional videos and educational modules to explain, for instance, oral health complications commonly associated with dementia [91].

Although several studies reported consistently high satisfaction levels among caregivers using these mOralHealth applications, they also noted that these materials are limited in their capacity for cultural adaptation, hence diminishing their impact on diverse populations [92]. Some applications additionally allow caregivers to document and monitor daily oral hygiene routines, including the condition and maintenance of dental prostheses, changes in oral health conditions, and adherence to recommended care. These functionalities may facilitate communication between caregivers and oral health professionals by providing structured records and follow-up information that can be shared among members of the care team [93].

One of the few mOralHealth tools developed specifically for dementia caregiving is Dental.Aging.Tips, a free Progressive Web App created at the University of Iowa for family and paid caregivers of persons living with dementia (Table 1). The platform provides evidence-based guidance on daily oral care, management of behavioral barriers, and recognition of common oral health problems [94,95]. In a pilot study involving 31 caregivers, significant improvements were observed in oral health knowledge, attitudes toward oral hygiene provision, and confidence in identifying oral health problems (all p<0.05), while paid caregivers also reported increased perceived support availability (p<0.001). Users highlighted its ease of navigation and practical value for denture care, lesion recognition, and management of care-resistant behaviors. Although clinical outcome data are not yet available, Dental.Aging.Tips has been recognized for integrating AI-based assistance features for caregivers of people with dementia [95,96].

A related caregiver-centered application is GeriaDental, a free mobile app for iOS and Android developed at the University of Iowa College of Dentistry to support caregivers and care staff responsible for older adults (Table 1). The app provides oral health education and practical guidance, with particular emphasis on managing oral care for individuals with cognitive impairment or mental health conditions [97]. GeriaDental represents an early initiative in the development of caregiver-focused mOralHealth technologies and served as a precursor to the later Dental.Aging.Tips platform. However, formal evaluations of its usability, acceptability, or clinical effectiveness have not yet been reported in the peer-reviewed literature.

Beyond educational interventions, some applications have been developed to support caregivers and nursing personnel in oral health assessment. One example is the digital Mini Dental Assessment (MDA), a tablet-based tool designed to enable non-dental personnel to perform standardized oral health screening in older adults (Table 1). The application integrates a chewing efficiency test, photographic reference scales, automated scoring, and structured data collection, reducing errors commonly associated with paper-based assessments [98]. Usability was exceptionally high (SUS score: 95.18±4.26), while interobserver reliability exceeded 0.93 across dentists, nurses, and non-medical evaluators, indicating that screening procedures could be performed consistently by non-expert personnel. These findings highlight the potential of mobile technologies to support caregiver and nursing staff training and facilitate the incorporation of oral health monitoring into routine geriatric care workflows [98].

Other applications incorporated reminder systems for oral hygiene routines and dental appointments, symptom-monitoring alerts, and, in some cases, options for synchronous or asynchronous teleconsultation. Although these features extend the functionality of mobile health tools beyond caregiver education, their integration into routine healthcare services remains inconsistent [36]. The studies reviewed indicate that greater engagement with these technologies was generally associated with better oral health outcomes, with frequent users showing larger improvements than occasional users [65]. Similarly, teleconsultation-based approaches appeared to be most successful when supported by established referral pathways, interdisciplinary collaboration, and sustained institutional infrastructure [99].

### 3.4. Mobile Applications for Training Primary Healthcare Personnel and Dental Providers

A fourth category comprises applications designed to train healthcare providers, particularly those working with older adults in community or institutional settings [100]. These tools typically provide content on geriatric oral health, clinical assessment practices, and preventive care, often incorporating interactive learning components such as quizzes, thematic modules, and case-based scenarios [86,93].

A few applications integrate clinical decision-support features to assist providers in identifying oral health risks, determining when referrals are needed, and supporting preventive interventions in older adults [101]. Such tools may be particularly useful in regions with limited access to specialized dental services, where they can support frontline healthcare personnel in addressing oral health needs within their scope of practice.

Other applications support community-level assessment through the digital collection of oral health indicators, facilitating screening activities in primary care and the implementation of large-scale oral health surveys [41]. These capabilities may be valuable for public health programs, as the resulting data can be incorporated into surveillance systems to monitor oral health trends and inform population-level interventions.

ToothPortal is a teledentistry platform developed by the University of British Columbia (UBC) that enables caregivers, nurses, physicians, patients, and dental professionals to securely share clinical information, photographs, and oral health records with a dental team for remote evaluation and triage (Table 1). Designed primarily to improve access to dental care for older adults living in long-term care facilities, the platform also serves as a training resource for healthcare providers and dental students involved in geriatric oral healthcare [102]. Developed within the UBC Geriatric Dentistry Program, ToothPortal is currently being implemented in long-term care settings in Vancouver, where supervised dental students use the platform to conduct remote oral health assessments and participate in clinical decision-making before face-to-face care [103]. Although formal evaluations of its effectiveness have not yet been published, the platform illustrates how teledentistry can simultaneously support workforce training and improve access to oral healthcare for institutionalized older adults.

### 3.5. Evaluation of Effectiveness, Acceptability, and Usability

Evaluations of mobile applications have shown considerable variability in both scope and methodological rigor. Particularly informative were studies that incorporated feedback from users and caregivers through surveys and usability assessments. Although these evaluations contributed to the refinement of existing applications, only a limited number of studies adopted formal co-design methodologies, and even fewer included participants from diverse cultural and socioeconomic backgrounds or with varying levels of cognitive function [34,86,104].

Older adults are often challenged when adopting new technologies, and this remains a significant barrier to mHealth implementation. Commonly reported obstacles include limited digital literacy, visual or motor impairments, difficulties in capturing clear intraoral images, and complex or non-intuitive user interfaces [63,89].

Another common limitation in the evaluation of mOralHealth technologies is the reliance on self-reported outcome measures, such as satisfaction questionnaires and usability scales. These instruments require a degree of cognitive and linguistic ability that may not be present among some dependent older adults or individuals with cognitive impairment, potentially limiting the representativeness of the findings.

This limitation was highlighted in the French e-DENT teledentistry project, where many intended users—including dependent older adults and individuals with cognitive disabilities—were unable to reliably complete conventional acceptability assessments. Researchers found that negative reactions were often related not only to the digital technology itself but also to anxiety or discomfort associated with dental procedures. These findings suggest that user experience in mOralHealth is influenced by both the technology and the clinical context in which it is used, underscoring the need for complementary observational methods when evaluating applications intended for functionally or cognitively impaired populations [105].

One example of a formally evaluated intervention is the MelloVR virtual reality relaxation application, assessed by Lahti et al. in a RCT involving 255 adult dental patients in Finland. Participants exposed to immersive 360° nature environments before treatment experienced significantly greater reductions in overall and anticipatory dental anxiety than those receiving usual care (β=−0.75, p<0.001 and β=−0.43, p<0.001, respectively). Acceptability was also high, with 98% of participants in a preceding pilot study describing the experience as relaxing and 87% indicating willingness to use virtual reality during future dental procedures. Although not specifically developed for older adults, these findings suggest that immersive digital technologies may represent a feasible and well-accepted approach for reducing anxiety associated with dental care [106].

A recent contribution to this area was provided by Muñoz-Sepúlveda et al. (2026), who evaluated a WhatsApp-supported teledentistry intervention among 120 older adults residing in rural and urban communities in southern Chile [107]. The intervention combined face-to-face oral health education with four theory-based educational videos delivered through WhatsApp, while the control group received conventional in-person education alone. Both groups showed substantial improvements in oral health knowledge and self-efficacy; however, participants receiving the telehealth reinforcement demonstrated significantly greater improvements in attitudes toward oral health (adjusted mean difference = 0.91, p=0.002), with effects remaining consistent across sensitivity analyses. Acceptability was high, with 92% of participants indicating that they would recommend the intervention to others. Notably, the largest benefits were observed among rural participants, suggesting that familiar and widely available communication platforms may help extend oral health promotion efforts to underserved populations. However, approximately one quarter of otherwise eligible individuals were excluded because they were unable to use WhatsApp, highlighting persistent digital literacy barriers among older adults despite the simplicity of the platform [107].

Overall, the acceptability and usability of mOralHealth applications appear to depend on both design characteristics and user expectations. Across the studies reviewed, older adults generally preferred applications that complemented, rather than replaced, interactions with healthcare professionals, highlighting the importance of integrating digital tools into existing care pathways. Factors consistently associated with user acceptance included content personalization, visual presentation of oral conditions, and interactive features. Considerable variation was also observed in users’ information preferences: while some valued detailed explanations to better understand their oral health, others found large amounts of information difficult to navigate or unnecessary [67]. Taken together, these findings reflect the diversity of needs and expectations among potential users of mOralHealth technologies and the importance of considering these differences when interpreting acceptability and usability outcomes.

### 3.6. Gaps Identified in the Literature

Several important gaps were identified in the current landscape of mOralHealth research and development. One notable gap is the small number of applications specifically designed for informal caregivers. Although some tools provide indirect support for caregiving activities, few have been intentionally developed to address the daily challenges faced by caregivers of older adults [92].

Another major gap is the scarcity of clinically validated interventions. While many applications report good usability and user satisfaction, relatively few studies have demonstrated measurable clinical improvements or evaluated long-term oral health outcomes. As a result, evidence regarding their effectiveness remains relatively weak, making it difficult to support their incorporation into clinical guidelines or public health programs [34,108].

Accessibility for older adults with cognitive impairment also remains limited. Only a small number of applications incorporate features tailored to individuals with dementia, mild cognitive impairment, or frailty, despite the high prevalence of these conditions in aging populations. This restricts the applicability of many mHealth tools among those who may benefit from them most [86,93].

A further gap is the lack of long-term evaluation. Few studies include extended follow-up periods, assessments of sustained adherence, cost-effectiveness analyses, or evaluations of how these tools are integrated into primary care, dental services, or community-based programs. Consequently, evidence regarding long-term effectiveness and real-world implementation remains scarce [86,93].

Another gap identified in the literature is the limited cultural and linguistic adaptation of mOralHealth applications. Most available tools have been developed and evaluated in a relatively small number of countries and languages, predominantly in English. This may limit adoption, usability, and sustained engagement among older adults and caregivers from diverse sociocultural backgrounds [35,109].

Collectively, these findings highlight the diversity of available mOralHealth technologies, but also the methodological and implementation gaps that continue to limit the strength and generalizability of the evidence.

**Table 1:** Summary of mOralHealth applications identified in this review, including target users, functionalities, study design, and evidence base.

Product	Target Users	Main Purpose	Key Functionalities	Special Features/Limitations	Study Design	Outcome Type	Evidence Base	Regional Context
SECAT App (Saudi Electronic Caries Assessment Tool; Alayadi et al., 2025) [88].	Dental health providers for field and community-based assessment.	Community survey support.	WHO-based dental caries charting, DMFT index calculation; real-time synchronization; community survey support.	Offline functionality for remote/low-connectivity settings; developed in Arabic and English.	Mixed-methods feasibility study with user-centered design, usability testing, and expert evaluation.	Usability, user satisfaction, and heuristic evaluation.	Peer-reviewed feasibility and usability study.	Middle East (Saudi Arabia).
MAKAR App (MAR-integrated oral health education; Romalee et al., 2024) [61].	Community-dwelling older adults.	Deliver interactive oral health education on tool selection and use via mobile augmented reality (AR)	Interactive 3D animated models and AR-enhanced 2D images of oral care tools; QR-code content access; instructional flyers for home use; single 1-h guided session with option for independent post-session use.	First MAR-based oral health education intervention; Chinese-language educational content; single brief session (no structured home-use requirement); below-average usability scores.	Three-arm parallel randomized controlled trial (MAR, lecture-based, control); assessments at baseline and follow-up.	Patient-reported: oral health knowledge, self-efficacy, and usability; clinical: plaque score, Löe–Silness gingival index, and tongue coating index.	Peer-reviewed randomized controlled trial.	Asia (Taiwan).
Dental.Aging.Tips App (Ashida et al., 2024) [95].	Family and paid caregivers of persons living with dementia.	Support oral care in aging and dementia.	Evidence-based guidance, step-by-step oral care instructions, dementia-focused content.	English-language Progressive Web App; strong caregiver focus; limited long-term clinical validation; may require complementary in-person training.	Two-phase pilot study: stakeholder-engaged prototype refinement and pre-post caregiver evaluation.	Caregiver-reported: oral health knowledge, attitudes toward oral care, perceived support, and confidence in identifying oral health problems.	Peer-reviewed pilot study.	North America (USA).
ToothPortal (University of British Columbia, 2024) [103].	Caregivers, nurses, physicians, dental students and geriatric dentistry teams.	Teledentistry and remote consultation.	Upload intraoral images, clinical data sharing, professional review.	English-language institutional platform; secure upload of oral health photos, medical/dental history; structured digital triage; care planning and urgency determination; remote consultation between care teams.	Implementation experience in long-term care settings.	Not formally evaluated.	Implementation reports (no peer-reviewed evaluation identified).	North America (Canada).
TEGO App (Geriatric Dental Specialties Tele-platform; Beltrán et al., 2022) [64].	Spanish-speaking older adults and caregivers.	Oral health education, remote triage, and specialist consultation.	Educational modules, audiovisual content, interactive games.	Culturally adapted Spanish-language platform; limited geographic availability; Android only; restricted to enrolled users during pilot phase.	Pilot feasibility study with pre-post assessment and user satisfaction evaluation.	Patient-reported: oral health knowledge, usability, satisfaction, and willingness to recommend.	Peer-reviewed pilot feasibility study.	Latin America (Chile).
OHEMA (Oral Health Education Mobile App; Ki et al., 2021) [55].	Community-dwelling older adults.	Improve oral health and swallowing-related quality of life through mobile-delivered education.	Oral exercise videos, intraoral and extraoral massage, customized oral hygiene instruction, motivational trot-song video; supplementary printed workbook and poster.	Korean-language intervention; enables repeated, asynchronous self-learning via YouTube; 1:1 home visits by researcher; self-checklist for adherence monitoring.	Parallel-group randomized controlled trial with pre-post assessment.	Patient-reported: xerostomia and swallowing-related quality of life (SWAL-QoL); clinical: tongue pressure and salivary flow rate.	Peer-reviewed randomized controlled trial.	Asia (South Korea).
OSCA (Oral Self-Care App; Chang et al., 2021) [65].	Adults with periodontal disease.	Personalized oral self-care support.	Developed using the Behavior Change Wheel framework; evidence-based recommendations and personalized feedback.	Chinese-language application; focal areas highlighted by periodontist; educational videos on periodontitis; limited caregiver-specific features.	Parallel-group randomized controlled trial with 4–8 week follow-up.	Patient-reported: usability and user engagement; clinical: plaque control record (PCR).	Peer-reviewed randomized controlled trial.	Asia (Taiwan).
MDA (Mini Dental Assessment; Schmidt et al., 2021) [98].	Non-dental staff in geriatric care settings.	Screen oral health status and chewing efficiency in older patients to identify the need for dental referral.	Structured dental history questionnaire; automated score calculation; digital alternative to paper-based assessment.	German-language interface; two-step visual verification for comminution grading; offline operation; Android-only platform.	Mixed-methods pilot study with usability testing, interobserver reliability assessment, and comparative clinical evaluation.	Usability, interobserver reliability, screening accuracy, and nurse acceptability.	Peer-reviewed pilot implementation study.	Europe (Germany).
MelloVR App (Lahti et al., 2020) [106].	Adult dental patients attending primary public dental care.	Reduce preoperative dental anxiety via short immersive VR relaxation in the waiting room.	Five 360° nature videos (beach, waterfall, underwater, space float, paddling; 1–3.5 min).	Language-independent immersive visual content; feasible in routine waiting-room setting; low cost; effects appeared stronger in women.	Parallel-group randomized controlled trial with pre-post assessment.	Patient-reported: overall dental anxiety, anticipatory anxiety, and treatment-related anxiety.	Peer-reviewed randomized controlled trial.	Europe (Finland).
iGAM App (Tobias et al., 2020) [82].	Adult persons and caregivers.	Remote monitoring of gingival health.	Dental selfies, questionnaires, dentist feedback, educational content.	Available in Hebrew and English; requires adequate image-capture skills.	Development study with user-centered Agile approach and pilot usability testing.	Usability and user experience.	Peer-reviewed feasibility and usability study.	Middle East (Israel).
Tooth Memo (OHSMA; Detsomboonrat et al., 2019) [86].	Adults, caregivers, and dental professionals; lay users aged 15–80 years.	Oral health data recording and surveillance.	Dental history documentation, epidemiological data collection, automatic index calculation; compatible with iOS and Android platforms.	Thai-language platform; offline functionality, Excel export, saves 182 min vs. manual method per 103-patient survey; iOS login instability, no multi-user data sharing, and no iPad interface.	Cross-sectional comparative study with reliability assessment and user satisfaction survey.	Usability, user satisfaction, and interobserver reliability.	Peer-reviewed feasibility and usability study.	Asia (Thailand).
GeriaDental App (Marchini et al., 2019) [97].	Formal and informal caregivers of older adults.	Improve oral hygiene in older adults.	Expert oral hygiene tips from dentists and hygienists, behavior management techniques for older adults with cognitive impairment.	English-language application; includes physical assistive tools; limited published effectiveness data.	Application described in institutional reports.	Not formally evaluated.	No peer-reviewed evaluation identified.	North America (USA).
Tele Pack X teledentistry system (Queyroux et al., 2017) [84].	Older adults residing in nursing homes.	Remote diagnostic assessment of dental pathology, chewing ability, and prosthetic rehabilitation status.	Intraoral video recording by dental assistant.	Language-independent clinician-operated system; no physician involvement required; 100% backup success; faster than face-to-face assessment; asynchronous store-and-forward model.	Multicenter diagnostic accuracy study with crossover design.	Clinical: diagnostic accuracy for dental pathology, chewing ability, and prosthetic rehabilitation; patient-reported: acceptability.	Peer-reviewed diagnostic accuracy study.	Europe (France and Germany).
Brush DJ App (Underwood et al., 2015) [63].	General users including older adults.	Promote evidence-based oral hygiene behaviors; links to NHS oral health resources.	Timed brushing with music, reminders, appointment tracking.	Available in multiple languages; not specifically designed for older adults; widely adopted.	Cross-sectional user perception study with qualitative analysis	Patient-reported: oral hygiene behaviors, brushing duration, perceived oral cleanliness, and recommendation intention	Peer-reviewed usability study	Europe (United Kingdom).

## 4. Discussion

**Figure 4:**
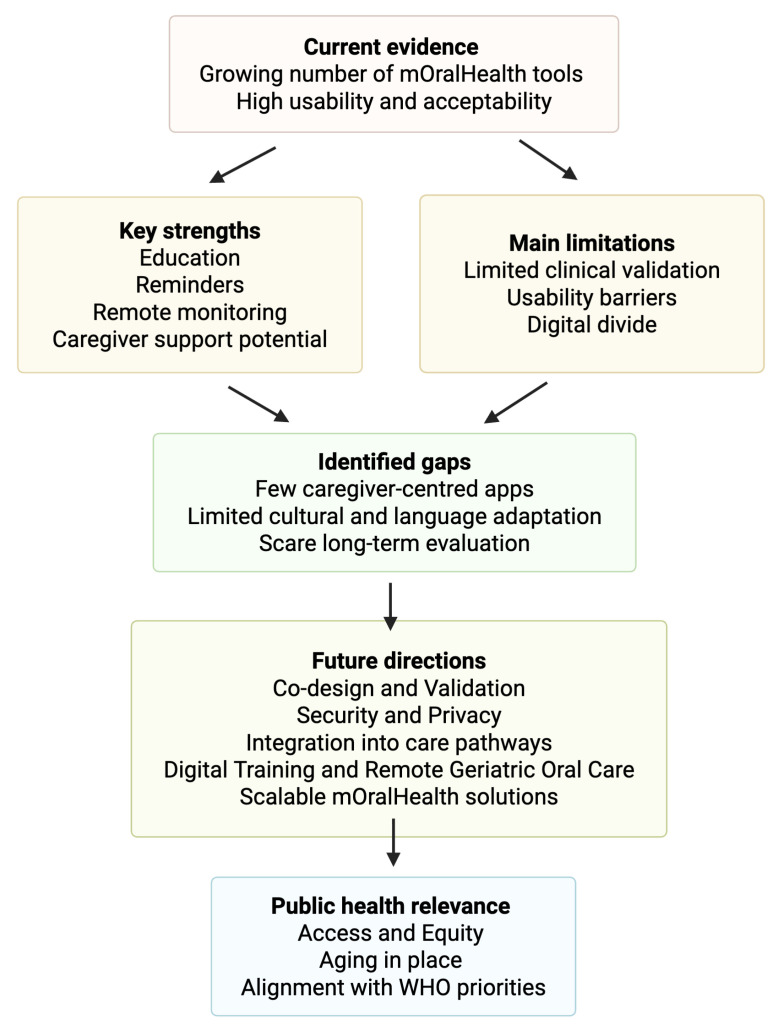
Summary of the main points discussed in this work. Created in https://BioRender.com.

This narrative review synthesized current evidence on mHealth applications developed for oral health promotion, monitoring, caregiver support, and professional training in the context of aging populations. The main points discussed in this section are summarized in Figure 4.

Our findings indicate that digital innovation in oral health is advancing rapidly. However, some of the specific needs of older adults and their caregivers, both formal and informal, remain insufficiently addressed. The wide range of available applications reflects the potential of mHealth to address important challenges in geriatric oral healthcare. Nevertheless, our review identified important limitations related to accessibility, clinical validation, and integration into routine care. We observed a growing use of mobile applications for oral health education and literacy. Common areas of focus include oral hygiene instruction [35,63,110,111], disease prevention [41,104,112], and oral health literacy [38,113].

Many applications seek to facilitate adoption among older adults through simplified navigation and multimodal educational formats, including text, videos, and interactive content [89,114,115]. However, most existing applications remain relatively generic and are rarely tailored to the clinical realities of aging. Older adults frequently experience complex oral health needs, including xerostomia, multimorbidity, prosthetic rehabilitation, and declining functional abilities. In addition, only a limited number of applications address cultural and linguistic diversity, which may restrict their use among non-English-speaking populations and contribute to persistent inequities in access to digital oral health resources [109]. This observation is consistent with a previous review published in 2023, which reported a marked geographical concentration of mOralHealth research in Asia, particularly in India [93]. The authors attributed this pattern to the widespread adoption of mobile technologies and the rapid expansion of digital health initiatives in the region.

Our findings similarly suggest that much of the available evidence originates from a relatively small number of countries, although studies from Europe, North America, and Latin America have begun to emerge (Table 1). Nevertheless, many interventions continue to be developed and evaluated within specific linguistic, cultural, and healthcare contexts, raising questions about their applicability to other populations. This issue is particularly relevant for older adults, whose patterns of technology use, health literacy, and access to care vary substantially across settings [116,117]. Greater attention to cultural adaptation, multilingual design, and local healthcare needs may therefore be necessary to ensure that mOralHealth technologies are relevant and accessible to diverse aging populations and their caregivers.

The limited number of applications developed specifically for Latin American populations is particularly noteworthy given the region’s demographic and healthcare context [118]. Latin America and the Caribbean are experiencing one of the fastest population aging processes worldwide, with adults aged 60 years and older expected to represent between 25% and 30% of the population by 2050 [119].

This rapid demographic transition leaves health systems with less time to adapt to the needs of an aging population [119]. At the same time, important barriers to oral healthcare persist across the region, including shortages of dental professionals in rural and peri-urban areas, a strong reliance on informal caregivers, and marked inequalities in digital literacy and access to mobile technologies [120,121,122]. In this context, the scarcity of locally developed and evaluated mOralHealth interventions represents an important gap in the current evidence base.

These inequalities are also reflected in access to digital technologies. Although mobile subscriber penetration reached 72% of the Latin American population in 2024 and access to mobile internet has expanded substantially over the last decade, an estimated 225 million people in the region still lacked mobile internet access at the end of 2023 [123]. Importantly, these connectivity gaps are concentrated in rural and peri-urban areas, where access to dental services is often limited and oral health needs are frequently greater. At the same time, these settings may offer important opportunities for mOralHealth interventions, particularly those focused on remote screening, monitoring, and early detection of oral health problems when face-to-face dental care is not readily available [107].

Applications designed for oral health monitoring and assessment represent one of the most promising areas of mOralHealth development, particularly those incorporating intraoral photography, self-examination guidance, and functional assessment tools [82,124,125]. However, their implementation in routine care remains limited by technological barriers and, perhaps more importantly, by the fact that most have been evaluated as stand-alone tools rather than as part of broader care pathways [64,126,127]. Emerging telehealth models suggest that some of these limitations can be addressed through greater integration with clinical services. For example, Iowa’s Virtual Dental Home (VDH) project linked dental hygienists working in long-term care facilities with remotely located dentists who provided diagnosis, triage, and treatment planning through an asynchronous teledentistry model [99].

The implications of these developments extend beyond patient care. Mobile applications, teleconsultation platforms, remote monitoring systems, and digital decision-support tools may also strengthen undergraduate and postgraduate training in geriatric dentistry by increasing exposure to homebound, institutionalized, and underserved older adults, populations that are often underrepresented in conventional clinical education. These technologies can facilitate caregiver training, interdisciplinary collaboration, and the management of medically complex older adults.

At the same time, relatively few applications were specifically designed to support the training of primary healthcare professionals in geriatric oral health [86,93,101]. This gap is particularly relevant in settings where access to specialists in geriatric dentistry is limited and primary care providers often serve as the first point of contact for older adults [99]. Integrating oral health modules into existing digital training platforms may represent a practical approach to strengthening workforce capacity and promoting the incorporation of oral health into primary care services [48]. As populations age and shortages of professionals with expertise in geriatric oral healthcare become more apparent, digital technologies may contribute not only to expanding access to care but also to preparing a workforce better equipped to meet the needs of older adults.

The limited number of applications specifically developed for caregivers deserves particular attention given their central role in supporting the daily oral care of dependent older adults. Although some tools include educational content, monitoring functions, or basic caregiver-oriented modules, few address the practical challenges commonly encountered in everyday care, such as managing resistance to oral hygiene, adapting care routines to cognitive impairment, or facilitating communication with healthcare professionals [48,128]. This gap is especially relevant in the context of population aging and the growing reliance on informal caregiving, where caregivers are increasingly expected to assume responsibilities for which they often receive limited training or support. The development of evidence-based digital tools that provide practical guidance, decision support, and timely access to professional advice may help strengthen caregiver capacity and improve oral health management among dependent older adults [129,130].

A persistent limitation of the mOralHealth literature is the scarcity of rigorous evaluation studies. Most publications focus on usability, user satisfaction, or application development, whereas relatively few assess clinical outcomes, behavioral change, long-term effectiveness, or implementation in routine care settings [131,132]. Randomized controlled trials, longitudinal studies, and real-world implementation research remain uncommon [133,134,135]. This limitation is reflected in a recent systematic assessment of oral hygiene applications using the Mobile Application Rating Scale (MARS), which reported a mean overall quality score of only 2.48 (SD = 0.77) across 21 commercially available apps [128]. Particularly concerning were the low scores for evidence-based content (mean = 1.8), as well as limited interactivity, minimal personalization, and interfaces that may be difficult to use for individuals with lower digital literacy or sensory impairments. Consequently, the evidence needed to support the recommendation and integration of specific mOralHealth applications into clinical practice, public health programs, or initiatives promoted by organizations such as WHO and PAHO remains limited.

Beyond questions of effectiveness and usability, the limited empirical validation identified in this review also raises important ethical considerations related to data privacy, informed consent, interoperability, and the use of algorithmic decision-support systems in geriatric oral healthcare [33,136]. As mobile applications increasingly collect information from older adults, caregivers, and healthcare providers, concerns regarding data governance and responsible use become particularly relevant in vulnerable populations [137]. Although artificial intelligence may support personalized guidance and clinical decision-making, questions related to transparency, bias, and accountability remain unresolved [138]. These considerations are especially important in geriatric oral health, where information is often incomplete or caregiver-mediated and digital tools are best viewed as complements to, rather than substitutes for, clinical judgment.

Persistent barriers to accessibility also warrant attention. Visual impairment, reduced dexterity, limited digital literacy, and cognitive decline may affect the ability of older adults to engage with mHealth technologies [139,140]. Despite this, relatively few studies reported the use of formal co-design approaches involving older adults or caregivers, and accessibility features such as voice assistance, adjustable text size, high-contrast displays, and simplified navigation were inconsistently implemented. Greater attention to these aspects will be needed to ensure that digital innovations remain accessible to the populations they are intended to serve.

From a public health perspective, the findings of this review are broadly consistent with current international priorities, including the WHO Global Strategy on Oral Health [48], the PAHO Regional Digital Health Strategy [2], the WHO mOralHealth programme [33], and the WHO Decade of Healthy Ageing 2021–2030 [47]. These initiatives emphasize the integration of oral health into primary care, the use of digital technologies to expand access to services, and support for caregivers and healthy aging. Although many of the applications identified in this review address one or more of these objectives, relatively few have been developed, evaluated, and implemented at a scale sufficient to support routine care.

The evidence reviewed suggests that mOralHealth technologies can contribute to oral health promotion, caregiver support, professional training, and remote service delivery for older adults [37,40,84]. However, the field remains characterized by fragmented development efforts, limited clinical validation, and uneven adaptation to the needs of diverse aging populations. Addressing these gaps will require collaboration among researchers, healthcare providers, policymakers, community organizations, and technology developers to ensure that future innovations are not only technically feasible, but also evidence-based, accessible, and responsive to local contexts [40,141,142,143].

## 5. Conclusions

This narrative review, conducted under the SANRA framework, identified five thematic areas in which mHealth applications have been developed to support oral health in aging populations. Although the number and diversity of available tools continue to grow, the evidence base remains limited. Most applications have been evaluated primarily through usability or feasibility studies, while only a small proportion have been assessed using controlled designs or objective clinical outcomes. Applications focused on oral health education and literacy constitute the most developed category, whereas important gaps remain in tools designed for informal caregivers, dependent older adults, and individuals with cognitive impairment.

The review also highlighted the limited availability of culturally and linguistically adapted applications, particularly for Latin America and other non-English-speaking settings. In addition, evidence regarding long-term effectiveness, real-world implementation, and integration into routine healthcare services remains scarce. As a result, many technologies have yet to demonstrate their impact on oral health outcomes at the population level.

Overall, mHealth technologies offer opportunities to support oral health promotion, self-care, caregiver assistance, professional training, and remote service delivery for older adults. However, their contribution to healthy aging will depend on the development of clinically validated, user-centered, and contextually relevant interventions that can be effectively integrated into primary care and community-based models of care, in alignment with the WHO Global Strategy on Oral Health and the WHO Decade of Healthy Ageing 2021–2030 [47,48].

Future work should emphasize interdisciplinary collaboration, equitable design, and rigorous evaluation to ensure that digital innovations remain accessible, relevant, and responsive to the needs of diverse aging populations and their caregivers.

## Data Availability

The original contributions presented in the study are included in the article; further inquiries can be directed to the corresponding authors.
